# Prognostic Utility of Prechemoradiotherapy Albumin-to-Alkaline Phosphatase Ratio in Unresectable Locally Advanced Pancreatic Carcinoma Patients

**DOI:** 10.1155/2021/6647145

**Published:** 2021-04-08

**Authors:** Veysel Haksoyler, Erkan Topkan

**Affiliations:** ^1^Medline Hospital, Clinics of Medical Oncology, Adana, Turkey; ^2^Baskent University Medical Faculty, Department of Radiation Oncology, Adana, Turkey

## Abstract

**Background:**

We investigated the prognostic usefulness of prechemoradiotherapy (CRT) albumin-to-alkaline phosphatase ratio (AAPR) in unresectable locally advanced pancreatic adenocarcinoma (LAPAC) patients managed with definitive concurrent CRT (CCRT).

**Methods:**

A sum of 136 LAPAC patients who consecutively underwent definitive CCRT was retrospectively analyzed. The AAPR (serum albumin (g/dL)/serum alkaline phosphatase (IU/L)) was calculated by using the parameters obtained from the routine biochemistry tests on the first day of the CCRT. Ideal AAPR cutoff was sought by utilizing receiver operating characteristic (ROC) curve analysis. The primary and secondary endpoints were the impact of the AAPR on the overall survival (OS) and progression-free survival (PFS) results, respectively.

**Results:**

At a median follow-up of 14.8 months (range: 3.2-85.7), the median PFS and OS times were 7.5 (95% confidence interval (CI): 6.0-9.0) and 14.9 months (95% CI: 11.9-17.9), respectively. The ideal common AAPR cutoff was identified at the rounded 0.46 (area under the curve: 72.3%; sensitivity: 71.2%; specificity: 70.3%) point that dichotomized the patients into two groups: low AAPR (L-AAPR; *N* = 71) and high AAPR (H-AAPR; *N* = 65) groups, respectively. Comparative survival analyses showed that the L-AAPR cohort had significantly shorter median PFS (6.8 (95% CI: 5.7-7.9) versus 11.3 (95% CI: 9.9-12.7) months; *P* = 0.005) and OS (12.8 (95% CI: 10.6-15.0) versus 19.2 (95% CI: 16.9-21.5) months; *P* = 0.001) durations than their H-AAPR counterparts, separately. Albeit the N1-2 (*P* = 0.004) and CA 19‐9 > 90 U/mL (*P* = 0.008) were also found to be associated with inferior outcomes, yet the results of the multivariate analyses ascertained the L-AAPR as an independent indicator of diminished PFS (*P* = 0.003) and OS (*P* = 0.002) results.

**Conclusion:**

The present results proposed that the pretreatment AAPR < 0.46 was a novel independent indicator of adverse PFS and OS in unresectable LAPAC patients undergoing definitive CCRT.

## 1. Introduction

Pancreatic adenocarcinoma (PAC) represents one of the poorest prognostic cancers with respective estimated median and 5-year overall survival (OS) rates of less than 12 months and 10% [1, 2]. Roughly 30% of all newly diagnosed PACs present with proven involvement of neighboring critical blood vessels, namely, unresectable locally advanced PAC (LAPAC) [3]. Chemotherapy alone, induction chemotherapy followed by radiotherapy (RT), and radical concurrent chemo-RT (CCRT) represent the broadly appreciated current treatment options for medically fit LAPAC patients [4–6]. However, comparable anticancer interventions in equivalent LAPAC stages may end up with significantly different clinical results. These critical contrasts are, to a large extent, related to the conventional use of the TNM (tumor-node-metastasis) staging system as the most trusted prognostic tool in such patients, which dismisses the substantial tumor- and host-related biological differences by depending solely upon the local and regional expansions of the index LAPAC. Therefore, such enormous contrasts in the same stage after equivalent treatments undoubtedly emphasize the pressing need for the discovery of novel biomarkers for better prognostic stratification of such patients, as these markers may play critical roles in radioresistance and chemoresistance.

Applauding their requisite functions in the initiation, progression, and dissemination steps of PACs, several blood-borne biomarkers have been diligently scrutinized to stratify these patients into significantly discrete prognostic groups [7–17]. In this regard, serum albumin (ALB) and alkaline phosphatase (ALP), which are readily attainable from regular biochemistry tests, represent two estimable biomarkers regarding their ample capacity to accurately reflect the actual biological and pathological alterations in the nutritional, inflammatory, and immune status of cancer patients [18–23]. To be specific, lower ALB and higher ALP levels, which are well-recognized facilitators of cancer growth and dissemination, are firmly linked to worse nutrition, depressed antitumoral immune response, and aggravated inflammatory status in cancer patients, including unresectable LAPACs [24–28]. Since past investigations exhibited that tumor-related detriments in the nutritional, antitumoral immune response and overall systemic inflammatory conditions were altogether correlated to tumor development and progression, the ALB-to-ALP ratio (AAPR) was postulated to be a reliable novel inflammation-based prognostic indicator in hepatocellular carcinoma in 2015 by Chan et al. [29]. Following this hypothesis-generating study, the prognostic worth of AAPR was further tested and invariably confirmed in numerous other tumor primaries including renal cell carcinoma [30], cholangiocarcinoma [31], upper tract urothelial carcinoma [32], non-small-cell and small-cell lung carcinoma [33–35], nasopharyngeal carcinoma [36], cervical carcinoma [37], breast carcinoma [38], and pancreatic ductal carcinomas [39] and pancreatic neuroendocrine tumors [40].

Although the prognostic worth of AAPR has been investigated in unresectable PACs undergoing exclusive chemotherapy by Zhang et al. [39], yet to our soundest information, the prognostic value of AAPR has never been examined in unresectable LAPACs treated with definitive CCRT before. In this manner, acknowledging the credible evidence in PACs, we aimed to retrospectively investigate the prognostic utility of pre-CCRT AAPR as a novel biomarker in unresectable LAPAC patients who received definitive CCRT.

## 2. Patients and Methods

### 2.1. Patient Population

A retrospective institutional database search was performed to identify all registered and pathologically verified unresectable LAPAC patients who received CCRT between January 2007 and June 2018 at Baskent University Medical Faculty. Unresectable LAPAC was defined as a primary tumor involving the celiac axis and/or superior mesenteric artery, namely, stage III (T_4_N_0-2_M_0_) disease per AJCC staging system (8^th^ ed.). Our standard diagnostic and staging workup for PAC patients was as previously reported elsewhere [16, 17, 41]. Briefly, all patients were carefully examined with abdominal magnetic resonance imaging (MRI), MR cholangiopancreatography, and endoscopic ultrasonography (if undergoing open abdominal exploration) for abdominal disease staging and chest computed tomography (CT) and brain MRI for the exclusion of lung/mediastinal and brain metastases, respectively. Each patient further underwent 18F-fluorodeoxyglucose- (FDG-) positron emission tomography- (PET-) CT for better exclusion of the possible systemic metastases. To be qualified for the study, the following requirements were additionally needed to be met by the patients: [1] aged between 18 and 80 years, [2] Eastern Cooperative Oncology Group (ECOG) performance status 0-1, [3] body mass index (BMI) > 18.5 kg/m^2^, [4] pathologically proven adenocarcinoma histology, [5] no history of chemotherapy or RT, [6] adequate pretreatment bone marrow function, [7] adequate hepatic and renal functions, [8] able to receive at least one cycle of concurrent chemotherapy during the abdominal RT course, [9] available chemotherapy and RT details, and [10] available follow-up clinical and radiological data.

### 2.2. Permissions, Consent, and Ethics

The retrospective study protocol was approved by the Institutional Ethical Committee of Baskent University Medical Faculty before the assortment of any patient data. Signed informed consent was provided by each patient or her/his legally charged delegates for the collection and analyses of blood samples and pathologic specimens for academic presentation and publication of the results in an anonymous fashion.

### 2.3. Concurrent Chemoradiotherapy

As reported in detail previously [16, 17, 41], all eligible patients underwent a radical CCRT protocol consisting of a total dose of 45 Gy RT (1.8 Gy/fraction/day) that encompassed the index LAPAC and involved nodes. Elective nodal irradiation was unpermitted per our institutional standards for newly diagnosed LAPACs. Each eligible patient received continuously infused 5-fluorouracil (225 mg/m^2^/day) throughout the RT course that was trailed by 2 to 6 courses of maintenance gemcitabine (1000 mg/m^2^ i.v. on days 1 and 8 every 21 days) at the discretion of the treating medical oncologists.

### 2.4. Albumin-to-Alkaline Phosphatase Ratio (AAPR) Measures

Pretreatment AAPR was calculated by using the serum measures of ALB and ALP obtained from the blood biochemistry tests on the first day of CCRT as AAPR = serum ALB (g/dL)/serum ALP (IU/L) according to Chan and colleagues' original definition [29].

### 2.5. Treatment Response Evaluation

Per our institutional follow-up norms for LAPAC cases, all patients went through intensive successive assessments every 3 months for the initial 2 years and at every 6 monthly intervals, or more frequently whenever necessitated, afterward. The first post-CCRT response assessment was performed at 3 months of CCRT by using restaging PET/CT and abdominal MRI scans per the EORTC 1999 guidelines' criteria [42]. Next, each patient was monitored every 3 months for the first 2 years and every 6 months after that time by total blood count and biochemistry tests, serum CA 19-9 concentrations, and PET/CT until the affirmation of a complete metabolic response and abdominal MRI scans were the preferred follow-up imaging tool in cases with affirmed complete metabolic response. Patients were further assessed with additional restaging tools, only if indicated.

### 2.6. Statistical Analysis

Our primary endpoint was the OS (interval between the first day of CCRT and the date of death/last follow-up), while progression-free survival (PFS: interval between the first day of CCRT and the date of any type of disease progression/death/last follow-up) comprised the secondary endpoint. Medians and ranges were utilized to describe continuous variables, whereas categorical variables were described by using frequency distributions. Intergroup correlative comparisons were performed by chi-square test, Student's *t*-test, Fisher's exact test, or Spearman correlation, as indicated. The accessibility of a pre-CCRT AAPR cutoff that may stratify the research population into two essentially distinctive OS and PFS outcomes was sought by using the receiver operating characteristic (ROC) curve analysis. Kaplan-Meier estimates and log-rank tests were used to reveal the potential influence of various risk factors on the OS and PFS outcomes. The potential interactions between the variables and survival results were assessed via utilizing the multivariate Cox proportional hazard model. Any two-sided *P* values < 0.05 were considered significant for intergroup comparisons.

## 3. Results

Our retrospective institutional data search revealed a sum of 136 patients who met the eligibility criteria for the present investigation. Pre-CCRT patient and disease characteristics were as displayed in Table 1. Median age was 58 years (range: 30-79) for the whole cohort. Most patients were male 105 (77.2%), with the pancreatic head being the commonest (*n* = 109; 80.5%) involved region. Sixty-nine (50.7%) patients had N0, while 36 (26.5%) and 31 (22.8%) patients were staged as N1 and N2, respectively. Per the landmark Charité Onkologie 001 (CONKO-001) randomized trial's critical CA 19-9 cutoff which was set at 90 U/mL [43], 97 patients (71.3%) had CA 19-9 measures higher than the critical value.

Thirty-six (26.5%) patients were still alive at a median follow-up of 14.8 months (range: 3.2-85.7), and 33 (24.3%) and 20 (14.7%) of them were locoregional recurrence and progression-free at the time of this final analysis, respectively. Distant metastases (DM) were the commonest cause of death which constituted 95 (95.0%) of all 100 deaths, while the respective 3 (3%) and 2 (2%) cases were reported to succumb directly due to uncontrolled local/regional primaries and comorbid conditions. For the entire cohort, the median and 5-year OS rates were 14.9 months (95% confidence interval (CI): 11.9-17.9) and 17.8%, individually, while the corresponding PFS rates were 7.5 months (95% CI: 6.0-9.0) and 11.1%, separately. Although all patients underwent comprehensive assessment for conversion surgery at the 6^th^ week of post-CCRT period, yet only 16 (11.8%) of them were judged to be well suited for this procedure, with R0 resection being successfully achieved in 12 cases.

**Figure 1:**
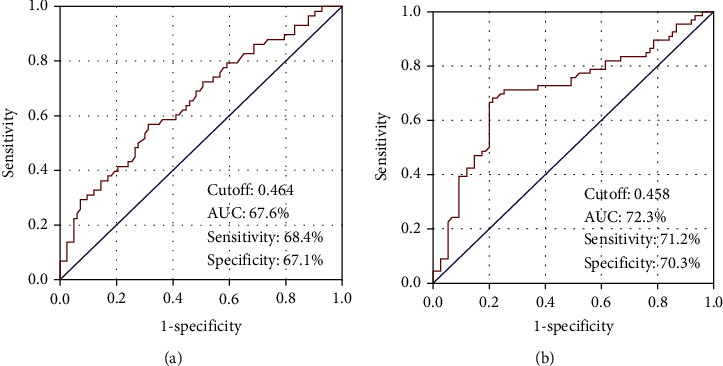
Results of receiver operating characteristic (ROC) curve analyses for (a) progression-free survival and (b) overall survival.

**Figure 2:**
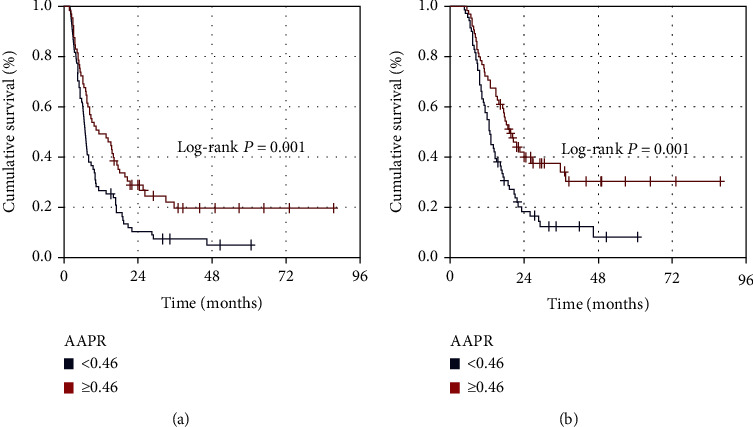
Survival outcomes per albumin-to-alkaline phosphatase ratio (AAPR): (a) progression-free survival and (b) overall survival (red line: AAPR ≥ 0.46; blue line: AAPR < 0.46).

To reveal the accessibility of the ideal pre-CCRT cutoff(s) of AAPR that interacts significantly with the clinical outcomes, we performed ROC curve analysis. The ideal AAPR cutoffs were determined to be 0.464 (area under the curve (AUC): 67.6%; sensitivity: 68.4%; specificity: 67.1%) for PFS and 0.458 (AUC: 72.3%; sensitivity: 71.2%; specificity: 70.3%) for OS, respectively (Figure 1). We utilized the rounded 0.46 as the common ideal cutoff to stratify patients into two groups for further intergroup comparisons: low AAPR group (L-AAPR: AAPR < 0.46 (*n* = 71)) and high AAPR group (H-AAPR: AAPR ≥ 0.46 (*n* = 65)), individually. Although the baseline demographics and patient characteristics were in general similar, the N1-2 (60.6% versus 36.9% for H-AAPR; *P* < 0.001) and CA 19‐9 > 90 U/mL (81.7% versus 60.1% for H-AAPR; *P* = 0.001) rates were significantly higher in the L-AAPR cohort. Survival analysis per AAPR group exhibited significantly inferior median PFS (6.8 (95% CI: 5.7-7.9) versus 11.3 (95% CI: 9.9-12.7) months; *P* = 0.005) and OS (12.8 (95% CI: 10.6-15.0) versus 19.2 (95% CI: 16.9-21.5) months; *P* = 0.001) times in the L-AAPR than the H-AAPR cohort, respectively. Additionally, suggesting the longer durability of the unfavorable survival rates in the L-AAPR group, the 5-year PFS (5.0% versus 19.6% for H-AAPR) and OS (8.3% versus 30.3% for H-AAPR) rates were likewise numerically inferior in the L-AAPR cohort (Figure 2).

In univariate analyses, we found that CA 19‐9 > 90 U/mL (versus ≤90 U/mL), N1-2 nodal stage (versus N0), and L-AAPR (versus H-AAPR) were the variables to reveal significantly inferior OS (*P* < 0.05, for each) and PFS (*P* < 0.05, for each) outcomes, separately (Table 2). As portrayed in Table 2, the results of multivariate analyses further ascertained that CA 19‐9 > 90 U/mL, N1-2 stage status, and L-AAPR status were independently associated with significantly unfavorable PFS (*P* < 0.05, for each) and OS (*P* < 0.05, for each) outcomes. Further comparisons between the groups at 5-year time point demonstrated that the CA 19‐9 > 90 U/mL (4.1% versus 18.9% for CA 19‐9 ≤ 90 U/mL), N1-2 nodal stage (4.2% versus 20.9% for N0), and L-AAPR (5.0% versus 19.6% for H-AAPR) patients had inferior OS rates than their counterparts with favorable features (Table 3).

## 4. Discussion

In our current investigation, contrasted to their H-AAPR counterparts, the unresectable LAPAC patients presenting with L-AAPR had poorer median and long-term PFS and OS independent of other confounding factors following conclusive CCRT. Thus, the present findings proposed that AAPR might be a novel biomarker in the dependable prognostic stratification of unresectable LAPAC patients undergoing radical CCRT when used in conjunction with other well-established prognostic factors.

Although our research results confirmed the well-recognized prognostic worth of the N stage and pretreatment CA 19-9 levels, the key finding of our exploration was the first time show of an independent prognostic incentive for pre-CCRT AAPR for unresectable LAPAC patients. Therefore, it is challenging to discuss our results and solidly deduce them in the apparent absence of similar research. Nevertheless, the outcomes introduced here still seem to be accordant with the accessible AAPR studies in other tumor primaries [29–38] and two respective postoperative [44] and systemic chemotherapy [39] series in unresectable PACs. The first study by Pu et al. [44] was a retrospective cohort analysis in 354 surgically resected PAC patients. In this study, the authors utilized the alkaline phosphatase-to-albumin ratio (APAR) rather than the AAPR and observed that patients with APAR > 2.16 (reversely corresponding to an AAPR of <0.46) had significantly shorter OS lengths than those with APAR > 2.16 (HR: 2.086; *P* = 0.004). The researchers additionally inferred that their nomogram consolidating the APAR to the AJCC framework was superior to the AJCC 8th ed. alone in terms of prediction of the postoperative clinical outcomes. More recently, Zhang et al. [39] in a group of 419 unresectable PAC patients treated with systemic chemotherapy reported that the AAPR ≤ 0.4 (versus AAPR > 0.4) was associated with significantly inferior median OS durations (6.4 versus 9.3 months; *P* < 0.001), which appeared to be an independent predictor of unfavorable OS in multivariate analyses (HR: 0.0556; *P* < 0.001). However, our current research differs remarkably from Zhang et al.'s investigation in at least two critical manners. First, although Zhang et al. practiced the unresectable LAPAC term, stage III patients constituted only 19.8% of their entire study population, unlike our 100% rate. And second, as all patients were treated with chemotherapy alone, the announced outcomes may not be reliably adapted to unresectable LAPAC patients undergoing definitive CCRT.

Other notable discoveries of our study were the finding that lower pretreatment AAPR values were linked to significantly higher CA 19-9 levels and higher rates of N1-2 status and DM. These discoveries are in good accordance with the previously mentioned Zhang et al.'s investigation, which showed altogether higher proportions of CA 19-9 levels and stage IV disease status in the L-AAPR than the H-AAPR gathering [39], as well as the investigations at other tumor sites exhibiting significantly higher N+ status in the L-AAPR cohorts [32, 45]. Further, strengthening these results, a recently published meta-analysis by Guo and colleagues demonstrated that lower AAPR values were associated with significantly increased rates of infiltration (*P* < 0.001), N+ status (*P* = 0.001), and DM (*P* = 0.028) rates [46].

Although the higher pre-CCRT rates of N1-2 and CA 19‐9 ≥ 90 U/m/L status and DM emergence during the follow-up period in the L-AAPR cohort may partly justify the inferior PFS and OS outcomes in this patient group, yet the elaborate mechanism(s) underlying the causal link between the L-AAPR and diminished survival times in LAPAC patients has not been comprehended to date. Nevertheless, some hypothetical yet sound judgments can be made by assessing the potential association between each key component of the AAPR formula and the clinical results. Low ALB levels represent inadequate nutritional and/or aggravated hypercatabolic states with resultant weight loss, which is a key determinant of cancer cachexia, a well-established poor prognostic factor for PAC patients [47]. Reduced ALB levels may furthermore reflect weakened antitumoral immunity, cellular phagocytic functions toward tumor cells, and antioxidant actions against the carcinogens, stimulated DNA repair and replication capacity of cancer cells, and decreased suppression of cell cycle and tumor progression [47, 48]. Low ALB levels may additionally indicate an aggravated inflammatory status as it is almost invariably connected with increased measures of C-reactive protein [23, 49]. Taken together, any impairment in ALB-mediated function may lead to the emergence of an extremely aggressive tumor phenotype characterized by enhanced tumor cell proliferation, growth, widespread metastasis, and resistance to anticancer treatments. Considering the ALP, decreased levels of ALP have been shown to suppress cell migration and induce cell death [23]. In contrast, ALP is recognized as a reliable cancer cell proliferation biomarker [22, 27], and its increased levels are associated with highly aggressive tumoral behavior and increased tumor burden and distant metastasis rates [50–52]. Although each of ALB and ALP has own prognostic value in various cancer types [53], yet AAPR has been shown to be a more powerful prognosticator than either of the ALB and ALP alone [29]. Furthermore, because the chance of being affected by various conditions like pregnancy, liver and bone diseases, fluid retention, and dehydration for the blend AAPR is less likely compared to its individual ALB and ALP components, it is sensible to anticipate that AAPR has a stronger and more dependable prognostic value in cancer patients, including the LAPAC cases as demonstrated here.

The present investigation is restricted by several drawbacks. First, it is a retrospective analysis with a relatively small cohort size. Therefore, our results should be interpreted with excessive caution till being confirmed by the results of appropriately designed large-scale prospective studies. Second, although ALB, ALP, and the resultant AAPR were fluctuating dynamic biomarkers, yet our AAPR values reflected just a single time point measure, which may have unpredictably altered the results in favor of one group. Accordingly, additional research explicitly addressing the AAPR dynamics throughout the disease course may aid usefully in the ascertainment of the most consistent time-dependent AAPR cutoff and its tangible consequences on clinical outcomes. Third, the results introduced here should not be generalized to all unresectable LAPACs, as we selected the patients with the fittest BMI (>18.5 kg/m^2^) and ECOG performance status (0-1). And fourth, the distinct adjuvant and/or salvage treatment preferences of the referring centers may likewise have modified the outcomes in a manner disproportionately favoring one group over the other one. However, despite these hindrances, because lower AAPR values symbolize either decreased ALB or increased ALP levels or both simultaneously, the present results and others from a rapidly growing number of tumor sites invariably imply that the easy to achieve, simple to calculate, and inexpensive AAPR might be utilized as a signifying biomarker of increased inflammation, decreased nutrition and antitumoral immunity, and increased resistance to treatment. Consequently, AAPR itself or nomograms consolidating AAPR may serve conveniently in the precise determination of the LAPAC groups with distinct prognoses and may enhance the wise selection of the best-fit individualized treatments.

## 5. Conclusions

The results of our retrospective cohort analysis demonstrated that the pretreatment AAPR < 0.46 was a sound and independent indicator of the grim prognosis for LAPAC patients who underwent definitive CCRT. Therefore, if confirmed by forthcoming large-scale studies, AAPR may serve conveniently as a reliable biomarker in the prognostic stratification and the vice selection of the most competent individualized treatment of such patients.

## Figures and Tables

**Table 1:** Baseline patient and disease characteristics for the entire study group and per low and high albumin-to-alkaline phosphatase ratio subgroups.

Characteristics	All patients (*N* = 136)	L-AAPR (*N* = 71)	H-AAPR (*N* = 65)	*P* value
Median age (years) (range)	59 (29-79)	59 (29-79)	58 (32-79)	0.86
*Age group,n* (%)				
<70 years	112 (82.4)	57 (80.3)	55 (84.6)	0.41
≥70 years	24 (17.6)	14 (19.7)	10 (15.4)	
*Gender,n* (%)				
Female	27 (19.9)	15 (21.1)	12 (19.4)	0.62
Male	109 (80.1)	56 (78.9)	53 (81.6)	
*ECOG performance,n* (%)				
0	73 (53.7)	36 (50.7)	37 (56.9)	0.54
1	63 (46.3)	35 (49.3)	28 (43.1)	
*Tumor location,n* (%)				
Head	112 (82.4)	58 (81.7)	54 (83.1)	0.72
Body/tail	24 (17.6)	13 (18.3)	11 (16.9)	
*N stage,n* (%)				
0	69 (50.7)	28 (39.4)	41 (63.1)	<0.001
1-2	67 (49.3)	43 (60.6)	24 (36.9)	
*CA 19-9,n* (%)				
≤90 U/mL	39 (28.7)	13 (18.3)	26 (28.8)	0.001
>90 U/mL	97 (71.3)	58 (81.7)	39 (60.1)	

Abbreviations: AAPR: albumin-to-alkaline phosphatase ratio; L-AAPR: low AAPR (<0.46); H-AAPR: high AAPR (≥0.46); ECOG: Eastern Cooperative Oncology Group; N stage: nodal stage; CA 19-9: cancer antigen 19-9; ALP: alkaline phosphatase.

**Table 2:** Outcomes of uni- and multivariate analyses.

Factor	PFS			OS		
	Univariate *P* value	Multivariate *P* value	HR	Univariate *P* value	Multivariate *P* value	HR
Age group (<70 vs. ≥70 years)	0.39	—	—	0.43	—	—
Gender (female vs. male)	0.65	—	—	0.78	—	—
ECOG (0 vs. 1)	0.82	—	—	0.80	—	—
Tumor location (H vs. B/T)	0.67	—	—	0.79	—	—
N stage (0 vs. 1-2)	0.003	0.006	1.87	0.004	0.005	1.78
CA 19-9 (<90 vs. ≥90 U/m/L)	0.006	0.009	1.56	0.008	0.012	1.48
AAPR (<0.46 vs. ≥0.46)	0.005	0.003	2.21	0.001	0.002	2.47

Abbreviations: PFS: progression-free survival; OS: overall survival; HR: hazard ratio; ECOG: Eastern Cooperative Oncology Group; H: head; B/T: body/tail; N stage: nodal stage; CA 19-9: cancer antigen 19-9; AAPR: albumin-to-alkaline phosphatase ratio.

**Table 3:** Median and 5-year survival results according to the factors demonstrating independent significance in multivariate analysis.

Survival	CA 19‐9 ≤ 90 U/m/L (*N* = 39)	CA 19‐9 > 90 U/mL (*N* = 97)	*P* value	N0 (*N* = 69)	N1-2 (*N* = 67)	*P* value	AAPR < 0.46 (*N* = 71)	AAPR ≥ 0.46 (*N* = 65)	*P* value
*OS*									
Median (mo.)	21.3	12.2	0.016	18.4	11.6	0.006	12.8	19.2	0.001
5-year (%)	28.7	6.3		26.1	10.4		8.3	30.3	
*PFS*									
Median (mo.)	13.4	5.6	0.022	12.2	6.3	0.003	6.8	11.3	0.005
5-year (%)	18.9	4.1		20.2	4.9		5.0	19.6	

Abbreviations: CA 19-9: cancer antigen 19-9; N0-2; nodal stage 0-2; AAPR: albumin-to-alkaline phosphatase ratio; OS: overall survival; PFS: progression-free survival.

## Data Availability

Data cannot be shared publicly because the data is owned and saved by Baskent University Medical Faculty. Data are available from the Baskent University Radiation Oncology Institutional Data Access/Ethics Committee (contact via Baskent University Ethics Committee) for researchers who meet the criteria for access to confidential data: contact address, adanabaskent@baskent.edu.tr.
